# LA67 Liposome-Loaded Thermo-Sensitive Hydrogel with Active Targeting for Efficient Treatment of Keloid via Peritumoral Injection

**DOI:** 10.3390/pharmaceutics15082157

**Published:** 2023-08-18

**Authors:** Hongshuang Wan, Shuangqing Wang, Chuying Li, Bowen Zeng, Hao Wu, Chao Liu, Liqing Chen, Mingji Jin, Wei Huang, Yingda Zang, Dongming Zhang, Zhonggao Gao, Zhehu Jin

**Affiliations:** 1 Keloid Research Center, Yanbian University Hospital, Yanji 133000, China; 2019001080@ybu.edu.cn (H.W.);; 2 State Key Laboratory of Bioactive Substance and Function of Natural Medicines, Institute of Materia Medica, Chinese Academy of Medical Sciences and Peking Union Medical College, Beijing 100050, China; 2021001114@ybu.edu.cn (S.W.);; 3 Beijing Key Laboratory of Drug Delivery Technology and Novel Formulations, Department of Pharmaceutics, Institute of Materia Medica, Chinese Academy of Medical Sciences and Peking Union Medical College, Beijing 100050, China; 4 Key Laboratory of Natural Medicines of the Changbai Mountain, Ministry of Education, College of Pharmacy, Yanbian University, Yanji 133000, China

**Keywords:** keloid, in situ hydrogel, pluronic, triptolide, Arg-Gly-Asp

## Abstract

A keloid is a benign tumor manifested as abnormal fibroplasia on the surface of the skin. Curing keloids has become a major clinical challenge, and searching for new treatments and medications has become critical. In this study, we developed a LA67 liposome-loaded thermo-sensitive hydrogel (LA67-RL-Gel) with active targeting for treating keloids via peritumoral injection and explored the anti-keloid mechanism. Firstly, Arg-Gly-Asp (RGD) peptide-modified liposomes (LA67-RL) loaded with LA67 were prepared with a particle size of 105.9 nm and a Zeta potential of −27.4 mV, and an encapsulation efficiency of 89.6 ± 3.7%. We then constructed a thermo-sensitive hydrogel loaded with LA67-RL by poloxamer 407 and 188. The formulation was optimized through the Box–Behnken design, where the impact of the proportion of the ingredients on the quality of the hydrogel was evaluated entirely. The optimal formulation was 20.7% P407 and 2.1% P188, and the gelation time at 37 °C was 9.5 s. LA67-RL-Gel slowly released 92.2 ± 0.8% of LA67 at pH 6.5 PBS for 72 h. LA67-RL-Gel increased adhesion with KF cells; increased uptake; promoted KF cells apoptosis; inhibited cell proliferation; reduced α-SMA content; decreased collagen I, collagen III, and fibronectin deposition; inhibited angiogenesis; and modulated the keloid microenvironment, ultimately exerting anti-keloid effects. In summary, this simple, low-cost, and highly effective anti-keloid liposome hydrogel provides a novel approach for treating keloids and deserves further development.

## 1. Introduction

A keloid is a fibrous proliferative disease, often secondary to skin trauma or inflammation, in which the scar gradually bulges, enlarges, infiltrates the surrounding tissue, and does not fade on its own, accompanied by itching, pain, and allergic symptoms [1]. When the area of the keloid is large, the patient’s movement in the affected area is restricted, bringing some psychological stress. As a common dermatological disease, the pathogenesis of keloids is not clear. Many studies have confirmed the association with elevated skin tension, hypoxia, chronic inflammation, genetics, autoimmune, and vascular factors [2], but effective treatment is lacking. Surgery, drug injections, laser, stress, and radiation therapy are standard therapies for the disease [3]. However, multiple local injections of hormones have adverse effects such as hypopigmentation, subcutaneous fat atrophy, capillary dilation, poor compliance, and susceptibility to infection. The surgical method suffers from a high recurrence rate. Radiation therapy carries a risk of carcinogenesis and is ineffective in treating large keloids. Patients treated with stress therapy have difficulty adhering to it for long periods; therefore, the results are less than ideal [4]. Thus, completely removing keloids has become a critical clinical challenge, and finding new treatments and medications has become crucial.

In recent years, natural products have proven to be remarkably effective in the treatment of challenging diseases [5,6]. Triptolide is a diterpenoid triepoxide extracted from Tripterygium wilfordii Hook. F. It is widely applied in China for the treatment of various autoimmune system diseases [7], cancer [8], viral infections [9], and fibrosis [10]. Triptolide has been found to inhibit the proliferation of fibroblasts and reduce the expression of connective tissue growth factor and platelet-derived growth factor, exerting a therapeutic effect on fibrosis [11]. However, triptolide is known to produce toxic side effects of liver and kidney damage [12]. As a triptolide derivative, LA67 was obtained by introducing a furoxan moiety into the 14-position of the triptolide core. LA67 was synthesized in the Institute of Materia Medica by Prof. Dongming Zhang [13]. LA67 maintains the activity of triptolide and can release nitric oxide, which improves the anti-tumor effect. LA67 was found to have a therapeutic impact on sepsis, colitis, melanoma, and colon cancer, with significantly reduced toxicity compared to triptolide [13]. Nevertheless, LA67 has the disadvantages of low solubility in water, low permeability, and poor bioavailability, which limit its further development.

Nanomedicine has been extensively applied to tumor treatment. The liposome (LNP) refers to a tiny capsule formed by the bilayer of lipids, which can encapsulate drugs within the phospholipid bilayer to form drug-carrying LNP, achieving the effects of increasing solubility, facilitating permeation, and reducing toxicity [14].

At present, there are some challenges in keloid therapy, mainly because the specificity of the keloid microenvironment is not fully utilized. Keloids have the characteristics of tumor tissue. There are a large number of capillaries at the junction of keloid and normal tissue, and vascular proliferation is obvious. Fibroblasts and neovascular endothelial cells at keloids have more abundant integrin receptors, which correlate strongly with unlimited cell proliferation. The Arg-Gly-Asp (RGD) peptide could specifically bind to integrin αvβ3 on the surface of cells, then bind to neuropilin-1 to enhance the penetration into the tissue via the C-end rule (CendR) [15]. In addition, cell adhesion ligands such as RGD peptides can be immobilized on nanocarriers to promote cellular uptake [16]. The exogenous RGD peptide is used for competitively binding the integrin receptor to inhibit fibroblast migration and neovascularization and simultaneously can be used for targeted drug delivery. RGD peptide is small in size, has a high affinity to integrin receptors, high stability, is easy to synthesize, and has no toxic effect [17]. Conjugating RGD to drug-loaded LNP could enhance therapeutic efficacy by enabling targeted delivery to keloid fibroblasts and neovascular endothelial cells.

Increasing the long-term effects of local treatment is also a vital issue to be solved. Injectable hydrogels have great potential as drug carriers in oncology treatment. Local delivery of therapeutic agents through hydrogels can provide more sustained release and high doses of medicinal drugs directly at the site of tumor tissue, reducing dosing frequency, preventing prolonged blood circulation, reducing side effects on normal tissue, and improving therapeutic outcomes of patients. Poloxamer hydrogel is a classical thermo-sensitive hydrogel system that has enabled the injectable [18], transdermal [19], mucosal adhesion [20], nasal [21], and ophthalmic [22] delivery of a variety of drugs such as curcumin [23], quercetin [24], resveratrol [25], and tannic acid [26]. Thermo-sensitive hydrogels remain liquid at room temperature to improve their injectability and diffusivity. They have the great potential to provide more effective and controllable release of therapeutics in response to changes in temperature. Therefore, this proven hydrogel is well-suited for treating keloids and is expected to be clinically applied soon. Yet, the gelation time of thermo-sensitive hydrogels is a crucial property affecting their clinical application, and the concentration and composition of the preparation material are the main factors of the gelation time. Therefore, it is necessary to screen the optimal hydrogel prescription to improve the therapeutic effect.

Therefore, this study prepared a thermo-sensitive hydrogel (LA67-RL-Gel) loaded with RGD-coated LA67 LNP for treating keloids via peritumoral injection. First, we prepared RGD-modified DSPE-PEG_2000_ (DSPE-PEG_2000_-RGD) using a maleimide bond. RGD-modified LNP loaded with LA67 (LA67-RL) was prepared by a thin film hydration method. Then, the formulation of the thermo-sensitive hydrogel was preliminarily optimized by the Box–Behnken design, where the impact of the proportion of the ingredients on the gelation time was evaluated entirely. Next, the effects of LA67-RL on proliferation, uptake, apoptosis, and migration of KF cells were evaluated at the cellular level. Finally, a keloid model of nude mice was established to assess the in vivo retention and anti-keloid effect of LA67-RL-Gel, and the anti-keloid mechanism was explored. We have innovatively prepared an LA67 LNP-loaded thermo-sensitive hydrogel with active targeting for the treatment of keloids with high precision and efficacy. Moreover, this simple, and low-cost LNP hydrogel is worthy of further development and industrial production.

## 2. Materials and Methods

### 2.1. Materials

DSPE-PEG_2000_-Mal was sourced from AVT (Shanghai) Pharmaceutical Tech Co., Ltd. (Shanghai, China). RGD was purchased from GL Biochem Ltd. (Shanghai, China). Cholesterol and coumarin-6 were obtained from J&K Scientific Co. Ltd. (Beijing, China). Soybean phospholipids (SPC) were purchased from AVT Shanghai Pharmaceutical Tech Co., Ltd. (Shanghai, China). Poloxamer 407 and Poloxamer 188 were obtained from Aladdin Biochemical Technology Co., Ltd. (Shanghai, China). The fetal bovine serum (FBS) and RPMI-1640 media were purchased from GIBCO, Invitrogen Corp. (Carlsbad, CA, USA). AnnexinV Alexa Fluor488/PI, PBS, and DAPI were purchased from Beijing Solarbio Science & Technology Co., Ltd. (Beijing, China). Cell counting kit-8 (CCK 8) was obtained from Dojindo Laboratories (Kumamoto, Japan). Roswell Park memorial institute-1640 (RPMI 1640) was obtained from Thermo Fisher Scientific Co., Ltd. (Beijing, China).

The keloid fibroblast (KF) cells were purchased from ATCC. The KF cells were cultured in RPMI-1640 supplemented with 10% (*v*/*v*) FBS, 1% (*v*/*v*) of a penicillin—streptomycin antibiotic blend, and 1% (*v*/*v*) glutamine. The cells were cultured in a humidified atmosphere at 37 °C and 5% CO_2_.

Male BALB/c nude mice (14–20 g) were obtained from Vital River Laboratory Animal Technology Co., Ltd. (Beijing, China). All animal experiments were approved by the Laboratory Animal Ethics Committee in the Institute of Materia Medica and Peking Union Medical College. All procedures followed ethical standards during the experiment.

### 2.2. Synthesis of Characterization of DSPE-PEG_2000_-RGD

The commercial DSPE-PEG_2000_-MAL (154 mg) and RGD (50 mg) were mixed in 10 mL of water using covalent bonding, stirred (600 rpm) at room temperature for 24 h, and the solution was transferred to a dialysis bag (MWCO 8–10 KDa, Sigma, Beijing, China) to purify the synthesized polymer. Dialysis was performed for a total of 3 d with water changes every 8 h. Finally, the modified polymer (DSPE-PEG_2000_-RGD) was obtained by freeze-drying.

The structure of DSPE-PEG_2000_-RGD was analyzed by an ^1^H NMR spectrometer instrument (400 MHz, Varian Inc., Troisdorf, Germany). The molecular structures of DSPE-PEG_2000_-Mal and DSPE-PEG_2000_-RGD were confirmed via nuclear magnetic resonance analysis. The molecular weights of DSPE-PEG_2000_-Mal and DSPE-PEG_2000_-RGD were detected by matrix-assisted laser desorption ionization time-of-flight mass spectrometry (Maldi-Top, METTLER TOLEDO, Zurich, Switzerland). A Bruker Alpha II spectrometer (Bruker Optik GmbH, Ettlingen, Germany) was used to obtain Fourier Transform infrared spectroscopy (FT-IR) spectra of DSPE-PEG_2000_-Mal and DSPE-PEG_2000_-RGD in the mid-infrared region (4000–400 cm^−1^).

### 2.3. Preparation of RGD-Modified LNP Loaded with LA67 (LA67-RL)

LA67-RL was prepared by a thin film hydration method [27]. Briefly, soy phosphate, cholesterol, DSPE-PEG_2000_-RGD, and LA67 were dissolved in chloroform. Then, the organic solvent was evaporated by rotary evaporation under a vacuum, and the film was formed and further dried using a vacuum drier. Then, 1 mL of PBS was added for hydration, and probe sonication (80 W, 10 s, 5 times) was performed to obtain LA67-RL. Meanwhile, the RGD-free-modified carrier LNP was noted as LA67-L.

### 2.4. Characterization of LA67-RL

Particle sizes, polydispersity indexes (PDI), and Zeta potential measurements were performed at 25 °C using a Zetasizer Nano ZS (Malvern, Worcestershire, UK). A Hitachi H-7650 Transmission Electron Microscopy was used to observe the morphology of LA67-RL.

Encapsulation efficiency (EE, %) of LA67-RL was detected by the direct method. Briefly, 200 μL of LA67-RL was mixed with 800 μL of acetonitrile and vortex sonicated to disrupt the structure of the LNP completely. The supernatant was obtained by centrifugation (14,000 rpm, 30 min). The drug concentration was determined by Agilent HPLC (Agilent 1260 Infinity; Agilent Technologies, Santa Clara, CA, USA), which was injected into an HPLC Allsphere ODS-2 (250 mm × 4.6 mm, 5 μm); the mobile phase was methanol:water = 70:30 at a flow rate of 1 mL/min under 35 °C column temperature, the injection volume was 10 μL, and the detection wavelength was 254 nm. The EE of the drug was calculated using Equation (1).

EE (%) = Weight of encapsulated drug/Weight of initial drug × 100%(1)

### 2.5. Preparation and Evaluation of Thermo-Sensitive Hydrogel

#### 2.5.1. Preparation of Thermo-Sensitive Hydrogel

A certain amount of P407 and P188 were added to 100 mL ultrapure water and stirred at 0 °C. After wholly dissolved, it was placed at 4 °C for standby.

#### 2.5.2. Determination of Gelation Time

The gelation time of the hydrogel was detected by the inversion method. P407 and P188 were fully mixed in different proportions. The penicillin vial containing 1 mL of the mixed solution was placed in a constant temperature water bath at 37 °C, and the changes in the mixture were observed. When the penicillin vial is inverted and the solution is no longer flowing, the solution is considered to have gelled, and the minimum time required is the gelation time of the thermo-sensitive hydrogel.

#### 2.5.3. Box–Behnken Design (BBD)

A BBD was applied to optimize the formulation of the thermo-sensitive hydrogel by Design Expert 11.1 software (Stat-Ease Inc., Minneapolis, MN, USA), and the aim was to research the influence of independent variables on response variables. The experimental design had two components as independent variables: P407 (%, X_1_) and P188 (g, X_2_). Gelation time (s, Y) was selected as a response variable to characterize the characteristics of the thermo-sensitive hydrogel.

According to the preliminary investigation results, the ranges of X_1_ and X_2_ were set, respectively, and the specific design is shown in Table 1. The design consisted of thirteen experimental runs to find the best-fit model and to evaluate the correlation between the independent variables and response variables (n = 3).

To adjust formulation, this study applied the following quadratic polynomial regression equations involving independent factors to model the impact of independent variables on the response and selected interactive elements for research based on model analysis, lack of fit, *p*-value, and R^2^ analysis of various measured responses. The effects of the independent variables X_1_ and X_2_ on Y used the quadratic mathematical model (Equation (2)):

Y = b_0_ + b_1_X_1_ + b_2_X_2_ + b_3_X_1_X_2_ + b_4_X_1_^2^ + b_5_X_2_^2^ (2)

where Y is the response, b_0_ is the intercept, and b_1–5_ are regression coefficients. X_1_ and X_2_ are individual effects. X_1_ and X_2_ are quadratic effects. X_1_X_2_ is the interaction effect. A one-way ANOVA was used to estimate the significance of the model (*p* < 0.05). Individual response parameters were evaluated using the F-test. The relationship between the corresponding and independent variable was analyzed using a connected three-dimensional graph.

#### 2.5.4. Preparation of LA67-RL-Gel

After the formulation of drug-free thermo-sensitive hydrogel was optimized, the LA67-RL-Gel hydrogel was prepared. Briefly, the LA67-RL was uniformly dispersed in the thermo-sensitive hydrogel system under ice-water bath conditions, and stirring was continued for 1 h to obtain the LA67-RL-Gel hydrogel finally. The LA67 concentration was 60 μg/mL. Meanwhile, RGD-free coated hydrogels were prepared and were denoted as LA67-L-Gel. The drug-free LNP hydrogels containing RGD modification were denoted as free RL-Gel.

#### 2.5.5. Surface Morphology of Hydrogels

The scanning electron microscope (Hitachi SU8010, Tokyo, Japan) was used to determine the surface morphology of the LA67-RL-Gel. The sample of lyophilized hydrogel was placed on a bronze rod containing double-sided tape, and gold was applied before evaluation.

#### 2.5.6. Rheological Properties

The sol-gel transition temperature and rheological properties of each examined formulation were tested using a Kinexus Lab+ rheometer (Malvern, UK). LA67-RL-Gel solution was cautiously applied to the lower plate of the rheometer, and equilibrated for at least 3 min before analysis, respectively. With the temperature being increased at 1 °C/min and within the temperature range of 15–45  °C, the strain amplitude values were acquired, respectively. The storage modulus (G′) and loss modulus (G′’) were measured.

### 2.6. Storage Stability

The long-term storage stability of LA67-RL and LA67-RL-Gel were investigated. Briefly, the LA67-RL and LA67-RL-Gel were sealed and stored at 4 °C, respectively. The appearance, particle size, and PDI were observed at 0, 1, 2, and 7 days, respectively.

The pH value of LA67-RL-Gel at 25 °C was measured at 0 and 7 days by a pH meter, respectively. Three samples were taken from each formulation for determination, and each sample was determined three times.

For the in vitro degradation experiment, the degradation of the LA67-RL-Gel was examined in pH 6.5 PBS at 37 °C at 0 and 7 days, respectively. The LA67-RL-Gel was taken out and lyophilized at different times.

### 2.7. In Vitro Release

The in vitro LA67 release properties of LA67-RL and LA67-RL-Gel were determined by the dialysis diffusion technique at pH 6.5 PBS. The 2 mL of LA67-RL suspension or 2 mL of LA67-RL-Gel was placed in a dialysis bag (MWCO 1 kDa, Sigma, Beijing, China) and then separately into the dissolution medium (10 mL pH 6.5 PBS). The dosage of LA67 was 120 μg. At 37 °C, 50 rpm, the sample (1 mL) was taken out at the desired time points, and the fresh solution was added. The LA67 concentration was measured using an HPLC.

### 2.8. Cell Research

#### 2.8.1. Cells Viability Assay

The in vitro cytotoxicity of the LA67-RL on KF cells was estimated using a CCK 8 assay. KF cells were plated into 96-well plates at a density of 6 × 10^3^ cells/well and were cultured for 24 h. KF cells were then treated with fresh medium containing serial concentrations of free LA67, LA67-L, and LA67-RL for 24 or 48 h of culture. CCK 8 reagent was added, and the cells were incubated for 1.5 h. Finally, the absorbance of the plates was measured at 450 nm using a Synergy H1 Microplate Reader (BioTek, Dallas, TX, USA) (n = 6).

Moreover, the CCK 8 assay was carried out to explore the in vitro biocompatibility of free-RL on KF cells for 24 and 48 h. The operational processes were identical to those of the in vitro cytotoxicity assays (n = 6).

#### 2.8.2. Cellular Uptake

The green fluorescent probe coumarin-6 (C6) labelled LPN with or without modified RGD (free C6, C6-L, and C6-RL) was prepared to visualize the cellular uptake and localization of LNP in KF cells. KF cells were seeded at the 12-well plates at a density of 1.5 × 10^5^ cells/well for 24 h. The fresh medium containing free C6, C6-L, and C6-RL at a C6 concentration of 2.5 µg/mL was added. After incubation for 5, 15, 60, and 120 min at 37 °C, respectively, the cells were washed three times and fixed with 4% paraformaldehyde for 10 min. The cell nuclei were stained with DAPI for 10 min. Then, the cells were washed three times. All the operations were kept in a dark place. Finally, the cells were observed and analyzed by confocal laser scanning microscopy (CLSM, TCS SP2; Leica, Germany).

To quantitatively investigate the cellular uptake efficiency of the LNP, flow cytometry analysis was used to estimate the fluorescence intensity of the stained cells. Briefly, KF cells were seeded in 6-well plates at a density of 2 × 10^5^ cells/well for 24 h. Then, the cells were incubated with a fresh medium containing free C6, C6-L, and C6-RL for 5, 15, 60, and 120 min at 37 °C, respectively. The concentration of C6 was 1 µg/mL. The cells were washed three times, collected by trypsinization and centrifugation, and resuspended in cold PBS. The fluorescence intensity of the cells was estimated by flow cytometry (Becton Dickinson, Franklin Lake, NJ, USA), with FlowJo 10.4 Software to deal with the data.

#### 2.8.3. Uptake Mechanism Study

To recognize the cellular internalization pathways of the LNP, the KF cells were cultured with different specific endocytic inhibitors, respectively, including RPMI 1640 (Control), methyl-β-cyclodextrin (M-β-CD, an inhibitor of lipid rafts, 5 mg/mL); sodium azide (NaN_3_, an inhibitor of mitochondrial respiratory chain complex IV, 3 mg/mL); chlorpromazine (an inhibitor of clathrin-mediated endocytosis, 10 µg/mL); chloroquine phosphate (an inhibitor of lysosomes, 5 μg/mL); colchicine (a tubulin inhibitor, 4 µg/mL); amiloride (a micropinocytosis inhibitor, 20 μg/mL); Brefeldin (an inhibitor of the ER-Golgi complex transport pathway, 25 μg/mL); Genistenin (27 μg/mL); and Monensin (an inhibitor of the Golgi complex-basolateral membrane pathway, 80 μg/mL) for 1 h at 37 °C. Subsequently, KF cells were incubated with C6-RL (10 μg/mL) for 2 h. The cells were then washed three times, trypsinized, collected, and measured by flow cytometry.

#### 2.8.4. Cell Apoptosis Assay

The anti-tumor activity of the LA67-RL was quantitatively analyzed using an Annexin V-FITC/PI apoptosis detection kit. KF cells were seeded in 12-well plates at a density of 2 × 10^5^ cells/well and were cultured for 24 h. The cells were treated for 24 h with free-L, free LA67, LA67-L, and LA67-RL (at a LA67 concentration of 1 μg/mL), with the complete medium as a control. The cells were collected by trypsinization and centrifugation, then resuspended in a binding buffer. Finally, 5 μL of annexin V-fluorescein isothiocyanate and 5 μL propidium iodide were added to the cells. The percentage of apoptotic cells was determined with flow cytometry.

#### 2.8.5. Cell Migration

The motility and migratory ability of KF cells after treatment with different formulations were estimated. KF cells were plated in 6-well plates at a density of 2 × 10^5^ cells/well and cultured until they formed a monolayer. A scratch wound was created in the middle of the well. The cells were washed to remove detached cells. The cells were treated with free LA67, LA67-L, and LA67-RL at a LA67 concentration of 1 μg/mL. Fresh medium was used as a control. Migrating cells in the wound area were observed and imaged at 0, 6, 24, and 36 h with an inverted light microscope (Olympus, Hamburg, Germany).

### 2.9. In Vivo Studies

#### 2.9.1. Establishment of Keloid Model

A mouse model of keloids was built by KF cells. KF cells in the logarithmic growth phase were digested with 0.25% trypsin, centrifuged, and precipitated. The cells were then resuspended in Matrigel^®^ (Corning, NY, USA), and the cell density was 1 × 10^7^ cells/mL and stored on ice. A total of 0.1 mL of the Matrigel^®^ prepared KF cells suspension was injected subcutaneously into the axilla of the right forelimb of the male BALB/c nude mice (14–18 g). And after 7 days, a keloid mass was seen subcutaneously in the right axilla of the mice. The size of the keloid was measured with a vernier caliper every two days to monitor the keloid volume. At the end of each experiment, the nude mice were humanely euthanized.

#### 2.9.2. In Vivo Retention Studies

First, the in vivo retention of the hydrogel was explored. Briefly, the fluorescent dye DiR was used to replace LA64 to obtain DiR-RL and DiR-RL-Gel. The hydrogel was injected subcutaneously next to the keloid using a 1 mL syringe at a DiR concentration of 10 μg/mL in a dose of 250 μL. The retention of the hydrogel was examined at the next 1, 2, 4, 6, 8, and 24 h using a small animal live imaging system. The excitation wavelength of DiR was 800 nm and the emission wavelength was 745 nm.

Next, the tissue distribution of DiR in vivo was examined. At 24 h, the nude mice were sacrificed and dissected to obtain keloids and major organs (heart, liver, spleen, lungs, and kidneys). The fluorescence intensity in different tissues was again observed by a small animal live imaging system and the fluorescence intensity was quantified.

#### 2.9.3. In Vivo Anti-Keloid Efficacy

When the keloid volume reached about 50 mm^3^, keloid-bearing nude mice were randomly divided into six groups (n = 6) as follows: normal saline, free LA67, LA67-L, LA67-RL, and LA67-RL-Gel at a dose of 0.8 mg/kg of LA67. The preparation was injected next to the keloid at a dose of 250 μL. The treatments were repeated every two days for eight times. Body weight and keloid volume were recorded every two days. During the seventeen days of treatment, the nude mice were euthanized, and the keloids were surgically removed. Apoptosis of cells was analyzed using the H&E and TUNEL assay. Keloids were fixed in 4% paraformaldehyde, embedded in paraffin, and cut into 4 μm thick sections. These sections were observed using light microscopy.

The expression levels of multiple proteins in keloids were evaluated by immunohistochemistry. Keloid sections were incubated with related primary antibodies at 4 °C overnight. After being rinsed three times, the sections were incubated with polymer-HRP secondary antibody (Klear Mouse DAB Kit, Golden Bridge International, Mukilteo, WA, USA) for 1 h at 25 °C. Immunoreactivity was revealed by the standard avidin–biotin immunoperoxidase method. Counterstaining with Meyer’s hematoxylin was then performed for 5 min. After that, they were evaluated under a light microscope (Olympus, Tokyo, Japan). The related primary antibodies were Ki 67 (0.5 µg/mL, ab15580), CD31 (1: 50, ab28364), Collage Ⅰ (1: 100, ab21286), Collage Ⅲ (1: 500, ab7778), Fibronectin (1: 50, ab2413), and α-SMA (1: 100, ab5694).

#### 2.9.4. In Vivo Safety

The blood biochemical analysis was performed for bio-safety evaluation, including creatinine (CREA), alanine aminotransferase (ALT), serum aspartate aminotransferase (AST), and blood urea nitrogen (BUN) [28]. The serum was separated by centrifugation at 4 °C, 4000 rpm. ALT, AST, BUN, and CREA were measured according to the instructions of the kit (Nanjing Jiancheng, China).

### 2.10. Statistical Analysis

All data are presented as the mean ± standard deviation. Statistical data comparisons among groups were performed by Student’s *t*-test or one-way ANOVA. Statistical analysis was performed using GraphPad prism 7.0 software. A *p*-value < 0.05 was regarded as statistically significant.

## 3. Results

### 3.1. Characterization of DSPE-PEG_2000_-RGD

**Figure 1:**
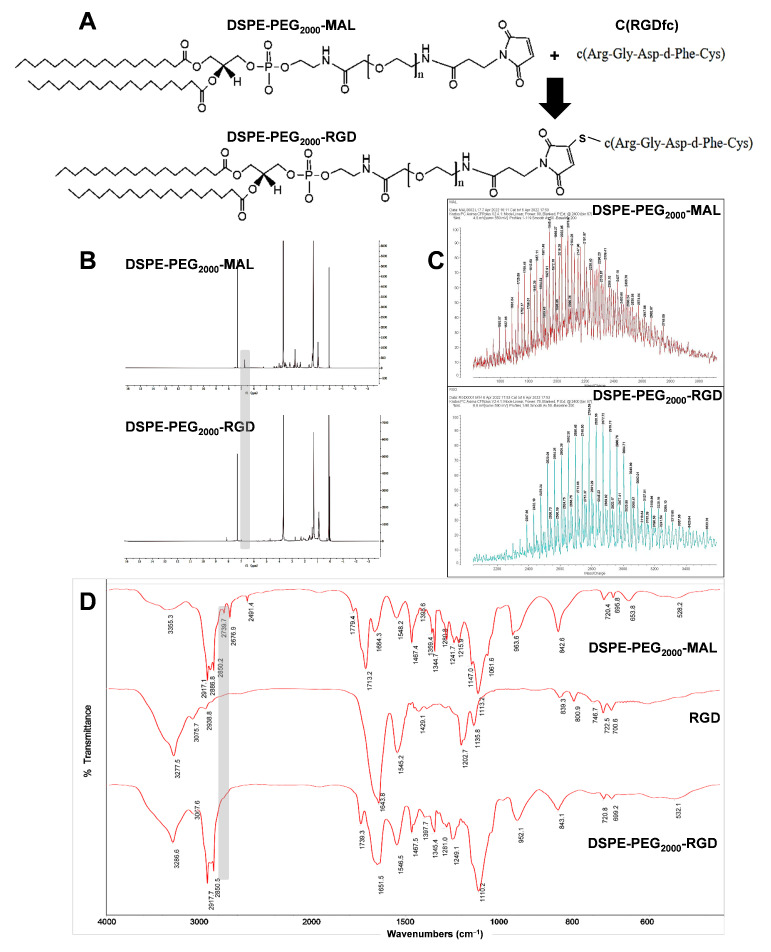
Synthesis and characterization of DSPE-PEG_2000_-RGD. (**A**) Synthesis route of DSPE-PEG_2000_-RGD. (**B**) ^1^H NMR spectra of DSPE-PEG_2000_-MAL and DSPE-PEG_2000_-RGD. (**C**) MALDI-TOF-MS spectra of DSPE-PEG_2000_-MAL and DSPE-PEG_2000_-RGD. (**D**) FT-IR spectra of DSPE-PEG_2000_-MAL, RGD, and DSPE-PEG_2000_-RGD.

DSPE-PEG_2000_-RGD was synthesized by the reaction of the maleimide group of DSPE-PEG_2000_-MAL and the sulfhydryl group of the RGD peptide, respectively. The synthesis reaction equation of DSPE-PEG_2000_-RGD is shown in Figure 1 A. In the ^1^H NMR spectrum of DSPE-PEG_2000_-MAL and DSPE-PEG_2000_-RGD (Figure 1B), signals that disappeared at δ = 6.7 (maleimide) confirmed their successful synthesis. The major peaks of 2077.0 and 2784.6 (Figure 1C) mass—charge ratios verified that the mean MWs of DSPE-PEG_2000_-MAL and DSPE-PEG_2000_-RGD were 2077.0 and 2784.6, respectively. Both agreed with their own calculated mean MW of DSPE-PEG_2000_-MAL and DSPE-PEG_2000_-RGD. The FT-IR results of DSPE-PEG_2000_-MAL showed that the characteristic absorption peak at 2886.8 cm^−1^ was –NH– in maleimide. The FT-IR results of DSPE-PEG_2000_-RGD showed that the characteristic peak at 2886.8 cm^−1^ disappeared, indicating that RGD was attached to the polymer and was relatively complete anyway. All of the above results indicated that DSPE-PEG_2000_-RGD was successfully prepared.

### 3.2. Characterization of LA67-RL

#### 3.2.1. Particle Size and Zeta Potential

**Figure 2:**
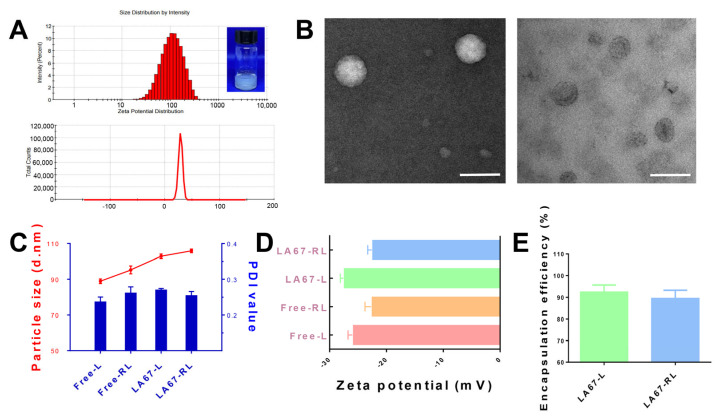
Characterization of LA67-RL. (**A**), Size distribution, PDI, and Zeta potential of LA67-RL by dynamic light-scattering analysis. (**B**) TEM images of LA67-RL (scale bar: 100 nm). (**C**) Particle size and PDI of free-L, free-RL, LA67-L, and LA67-RL (n = 3, means ± SD). (**D**) Zeta potential of free-L, free-RL, LA67-L, and LA67-RL (n = 3, means ± SD). (**E**) Encapsulation efficiency of LA67-L and LA67-RL (n = 3, means ± SD).

In order to improve the pharmacodynamic activity of LA67 on keloids, RGD-grafted and PEGylated lipid nanoparticles loaded with LA67 were successfully constructed. The particle size is a crucial determinant of the ability of nanoscale carriers to enter the tumor microenvironment through the EPR effect. LA67-RL has a particle size of 105.9 nm, a potential of −27.4 mV, and a PDI value of less than 0.3 (Figure 2A). LA67-L has a particle size of 102.9 nm and a potential of −22.4 mV. Although RGD had no significant effect on the particle size of LNP (Figure 2C), it decreased the potential (Figure 2D). This is because RGD carries a positive charge. The surface charge of LNP and cell membranes play a crucial in cell—LNP interactions [29]. Zeta potential is a key evaluation parameter for assessing the desired properties of LNP, which may affect in vivo stability and efficacy. DSPE-mPEG_2000_ is a negatively charged phospholipid. In addition, it is likely that the reduction of unconjugated maleimide to maleimic acid is responsible for the high negative Zeta potential of these preparations. The TEM image (Figure 2B) showed a uniform particle size of LA67-RL, consistent with the DLS measurement result. All these properties would be effective for LNP to gather at the tumor site.

#### 3.2.2. Encapsulation Efficiency (EE)

EE is a vital parameter to display the reasonability of LNP’s formulation and process. The higher EE showed that all of LNP had good drug loading efficiency. Compared to LA67-L, the EE of LA67-RL was lower, but EE was 89.6 ± 3.7% (Figure 2E). This phenomenon may be due to part of the lipid which was substituted by the DSPE-PEG_2000_-RGD, thus reducing the loading of the hydrophobic drug [30].

### 3.3. Preparation and Characterization of Hydrogel

#### 3.3.1. Box–Behnken Design (BBD)

The purpose of prescription optimization is usually to determine the level of variables in order to produce robust formulations with high quality characteristics. The thermo-sensitive hydrogels were prepared according to the table of BBD for each combination of horizontal factors and at different concentrations. Thermo-sensitive hydrogels were successfully prepared by a low-temperature stirring dissolution method, which is simple and the results are reproducible. The gelation time at 37 °C was the response value. Table 2 showed the specific values of the two response variables after the prescription design.

Y showed values in the range of 8.1–36.0 s. Although the experimental values span a wide range, they are usually acceptable. The effect of the independent variable on the response variable was studied and the results are presented in Equation (3).

Gelation time: Y = 9.60 + 2.50X_1_ − 0.20X_2_ − 0.75X_1_X_2_ + 12.45X_1_^2^ + 4.20X_2_^2^ (3)

**Figure 3:**
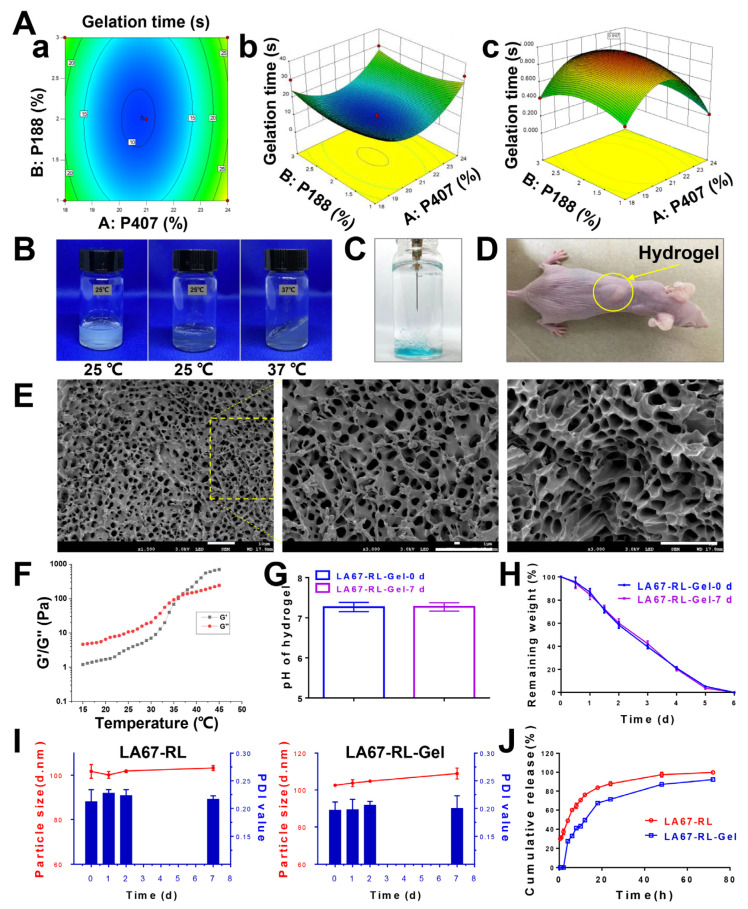
Characterization of LA67-RL-Gel. (**A**) The influence of independent variables on response variables. (**a**) contour plots, (**b**) 3-D response plots, and (**c**) 3-D response plot of the predicted theoretical values. (**B**) The phase transition photograph of the thermo-sensitive hydrogel. (**C**) Injectability of LA67-RL-Gel. The yellow circle showes the in vivo state of the hydrogel. (**D**) Hydrogel injected subcutaneously into nude mice. (**E**) SEM images of LA67-RL-Gel (scale bar: 10 μm). The dashed box indicates a localized enlargement of the hydrogel. (**F**) Rheological properties. (**G**) The pH of LA67-RL-Gel was at 0 and 7 days of storage (n = 3, means ± SD). (**H**) LA67-RL-Gel was degraded at 0 and 7 days of storage (n = 3, means ± SD). (**I**) The size stability and PDI stability of LA67-RL and LA67-RL-Gel for 7 days (n = 3, means ± SD). (**J**) The drug release behavior of LA67-RL and LA67-RL-Gel within 72 h (n = 6, means ± SD).

Considering the interaction of the two variables examined, we aimed to explain the magnitude of the coefficient of the effect of the parameters. The scale of each suggested regression coefficient was used to quantify each parameter’s impact on their responses. The positive algebraic sign positively influenced the responses, whereas the negative algebraic sign had an opposite effect on the responses. The analysis of the change results of the two response parameters is shown in Table 3, and the 3-D response surface plots are shown in Figure 3A(a,b). When *p* < 0.05, the established model is significant. The correlation coefficient (R^2^) represents the prediction accuracy of the fitted model. The larger the R^2^ of the model, the higher the precision of the model and the better the correlation. Considering the results for continuous *p*-values, lack of fitted *p*-values, SD, and R^2^ values, the quadratic model was the best-fitting model for the hydrogels.

ANOVA analysis was performed on the experimental data; the results are shown in Table 2. From the analysis in Table 3, it can be seen that the R^2^ of the model is 0.0069, which is less than 0.05, indicating that this experimental method is feasible. From the *p*-value, the secondary effect of P407 concentration significantly affected the gelation time of thermo-sensitive hydrogels (*p* < 0.001). The interaction effects have a negligible impact on the response values, demonstrating a weak interaction between the factors. Figure 3A(a,b) showed the contour plots and 3-D response plots of the interaction response surface plots of each factor. The highest points and contours of the response surface showed that extreme values existed for the two elements in the selected range, corresponding to the optimal conditions of 20.7% for P407 concentration, 2.1% for P188 concentration, and 9.5 s for gelation time.

#### 3.3.2. Feasibility Check

In order to test the feasibility of the method, the optimal conditions obtained by BBD were used to prepare thermo-sensitive hydrogels. The gelation time was measured at 37 °C, and the average value of three experiments was 10.5 ± 0.9 s, which was similar to the theoretical value. Figure 3A(c) showed the 3-D response plot of the predicted theoretical values. The results indicate that this response analysis method is feasible for the thermo-sensitive hydrogels prepared by P407 and P188. The compositional concentrations of P407 and P188 were optimized by response surface methodology to derive the optimal concentration of the thermo-sensitive hydrogel, which provides a theoretical and formulation basis for subsequent studies on treating keloids. It is worth noting that adding LNP did not significantly change the gelation time.

#### 3.3.3. Properties of Thermo-Sensitive Hydrogel

The in vivo temperature-sensitive behavior of thermo-sensitive hydrogels was first examined. Figure 3B shows the phase transition photograph of the thermo-sensitive hydrogel. The hydrogel appears as a solution at room temperature and a non-flowable gel at 37 °C. Figure 3C shows the injectability of the hydrogel. The hydrogel was injected subcutaneously into nude mice and the hydrogel solidified quickly (Figure 3D), demonstrating the hydrogel’s excellent in vivo temperature-sensitive behavior.

The microscopic morphology of the lyophilized hydrogel was observed by SEM (Figure 3E), and it was found that the surface of the hydrogel contained a large number of circular pore structures, which were relatively uniform and dense. The hydrogel had a moderate mechanical strength to resist compression during skin stretching.

The G′ and G″ both increased with the increase of temperature (Figure 3F), and the change rate of G′ was obviously greater than that of G″, which indicated that the gel solution gradually changed from a liquid state to a semi-solid state with the increase of temperature. When G′ and G″ are equal, the temperature at this time is the gelation temperature. The hydrogel formation temperature of LA67-RL-Gel was 36–37 °C. As the temperature continues to rise, G′ is always greater than G″. The gel adhered to the administration site in a semi-solid state, increasing local residence time.

### 3.4. Stability

The stability of preparation under biological conditions is a necessary parameter controlling the activity of the associated therapeutic agent. Stability studies of LA67-RL and LA67-RL-Gel were performed at 4 °C. The pH of LA67-RL-Gel was measured to be 7.3 ± 0.1 (Figure 3G), which was suitable for the human body. Therefore, the hydrogel can be used as a positive delivery system for local administration. The pH of the hydrogel did not change significantly after 7 days of storage.

The degradation profile of LA67-RL-Gel was determined by the membrane-less model and the results are shown in Figure 3H. The hydrogel was completely degraded at 6 h. The results showed that the thermo-sensitive hydrogel could form a drug reservoir in the keloid site, and play a sustained release function through slow degradation.

Within 7 days, all LA67-RL and LA67-RL-Gel solutions were clear, with no aggregation or precipitation. The particle size (103.3 and 108.9 nm) and PDI (0.2 and 0.2) of LA67-RL released from LA67-RL-Gel and LA67-RL did not change significantly by particle size measurements (Figure 3I). The storage stability of LA67-RL and LA67-RL-Gel was demonstrated. This stability could result from electrostatic repulsion between the negatively charged colloidal particles and also the phospholipid composition and drug to lipid ratio in this LNP. LNP was dispersed uniformly in the hydrogel of high molecular polymer, and the stability was further improved due to intermolecular hydrogen bonding.

### 3.5. In Vitro Release

The drug release behavior of LNP is an essential characteristic that will affect its delivery efficiency. LA67-RL-Gel slowly released 92.2 ± 0.8% of LA67 at pH 6.5 PBS for 72 h. The drug-release curves of LA67-RL and LA67-RL-Gel showed biphasic release (the initial rapid release and followed sustained release) of LA67 (Figure 3J). This early occurrence may be due to the LA67 being located on or near the surface of the LNP. As the diffusion distance between LNP and the release medium increased, the later release profile presented sustained release. The PEG coating may also create a more stable nanoparticle structure that could lead to a slower drug release. The presence of hydrogel further increased the distance of drug release and achieved the effect of sustained release.

### 3.6. Cell Researches

#### 3.6.1. Cytotoxicity

**Figure 4:**
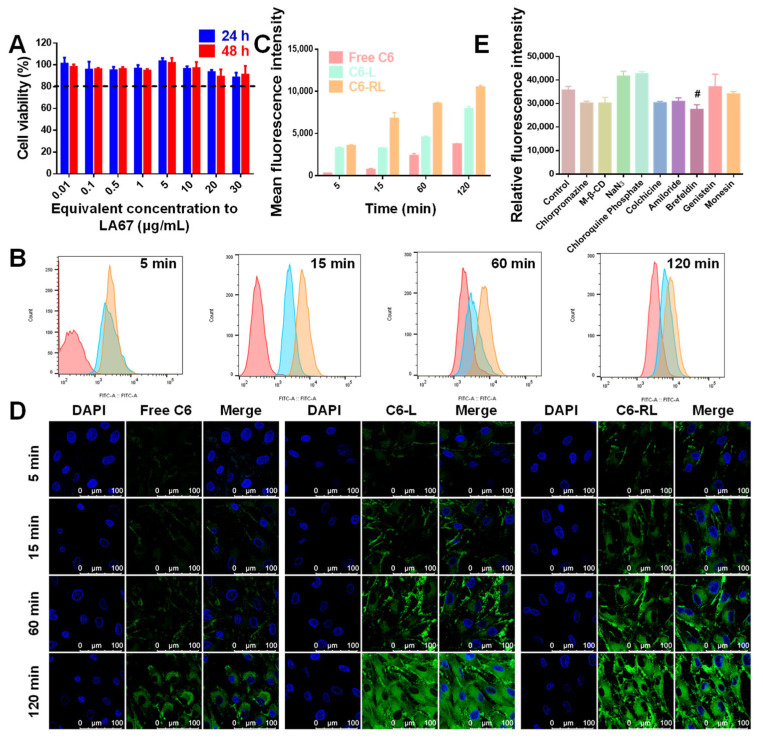
In vitro cytotoxicity and cellular uptake. (**A**) In vitro cytotoxicity of free-RL on KF cells after incubation for 24 and 48 h (n = 6, means ± SD). (**B**) Cellular uptake by flow cytometry after incubation with free C6, C6-L, and C6-RL for 5, 15, 60, and 120 min, respectively. (**C**) Statistical analysis of the data in (**B**) (n = 6, means ± SD). (**D**) CLSM observation of KF cells after treatment with free C6, C6-L, and C6-RL for 5, 15, 60, and 120 min (scale bar: 100 μm). (**E**) Cellular uptake analysis of C6-RL after incubation with different endocytic inhibitors by flow cytometry (n = 6, means ± SD), ^#^ *p* < 0.05.

To be useful, at the suitable concentration, preparation should be biocompatible, reduce the dosage required, and not influence the biological functions of cells. Thus, the cytotoxicity of free-RL was assessed using a CCK 8 assay in KF cells. When KF cells were incubated with a series of concentrations of free-RL equivalent to 0.01–30 μg/mL LA67 for 24 and 48 h, all groups exhibited low cytotoxicity with cell viability of more than 85% (Figure 4A). The results demonstrate that free-RL at predetermined concentrations and incubation times did not significantly influence the biological activity of KF cells.

#### 3.6.2. Cellular Uptake

Drug activity depends on drug transport to specific target sites and subsequent cellular uptake. We used C6 (a green fluorescent marker with a high fluorescent intensity and a low leaking rate) as a fluorescent marker. The uptake characteristics of LNP were studied by CLSM and flow cytometry. First, we investigated the time-dependent uptake of C6-RL by KF cells. C6-RL was co-incubated with KF cells for 5, 15, 60, and 120 min, and the cells were visualized by CLSM. As shown in Figure 4D, after co-incubation for 5 min, the C6-L and C6-RL groups showed that green fluorescence appeared inside the cells, indicating the entry of fluorescent nanoparticles into the cells. Next, the fluorescence intensity gradually increased with the increase of co-incubation time, and the strongest fluorescence intensity was observed at 120 min. However, the fluorescence intensity of the C6-RL group was stronger than that of C6-L in 5–60 min. At 120 min, the fluorescence intensities of the C6-L and C6-RL groups were the same. This is because of the saturation of cellular uptake at 120 min. The free C6 group consistently showed a relatively weak green fluorescence, indicating the lowest uptake. The flow cytometry results were consistent with the CLSM results (Figure 4B,C). These results suggest that LA67-RL facilitates cellular uptake of the drug, and the presence of RGD further enhances this effect.

#### 3.6.3. Uptake Mechanism

The intracellular mechanism of LA67-RL was studied by setting up a series of inhibitors to pre-incubate with KF cells, and the cellular uptake was marked according to the fluorescence intensity. This study also examined endocytosis pathways of C6-RL with KF cells. As shown in Figure 4E, various inhibitors, including Chlorpromazine, Methyl-β-CD, NaN_3_, Chloroquine Phosphate, Colchiccine, Amiloride, Brefeldin, Genistein, and Monensin, were added to the KF cells before treatment with C6-RL, separately. As shown in Figure 4E, when the KF cells were co-incubated with Chlorpromazine, Methyl-β-CD, NaN_3_, Chloroquine Phosphate, Colchiccine, Amiloride, Genistein, and Monensin, the uptake of KF cells did not decrease remarkably. When the cells were co-incubated with Brefeldin, LA67-RL uptake in KF cells decreased significantly. The uptake was reduced by 23.1 ± 5.5% times. The results demonstrated that the endoplasmic reticulum and Golgi apparatus are involved in the transport of nanocarriers into cells, and Brefeldin activates the GTP-exchange factors of Afr1p GTPase and inhibits LNP transport from the endoplasmic reticulum to the Golgi apparatus, which is a critical pathway involved in cell uptake.

#### 3.6.4. Cell Viability

**Figure 5:**
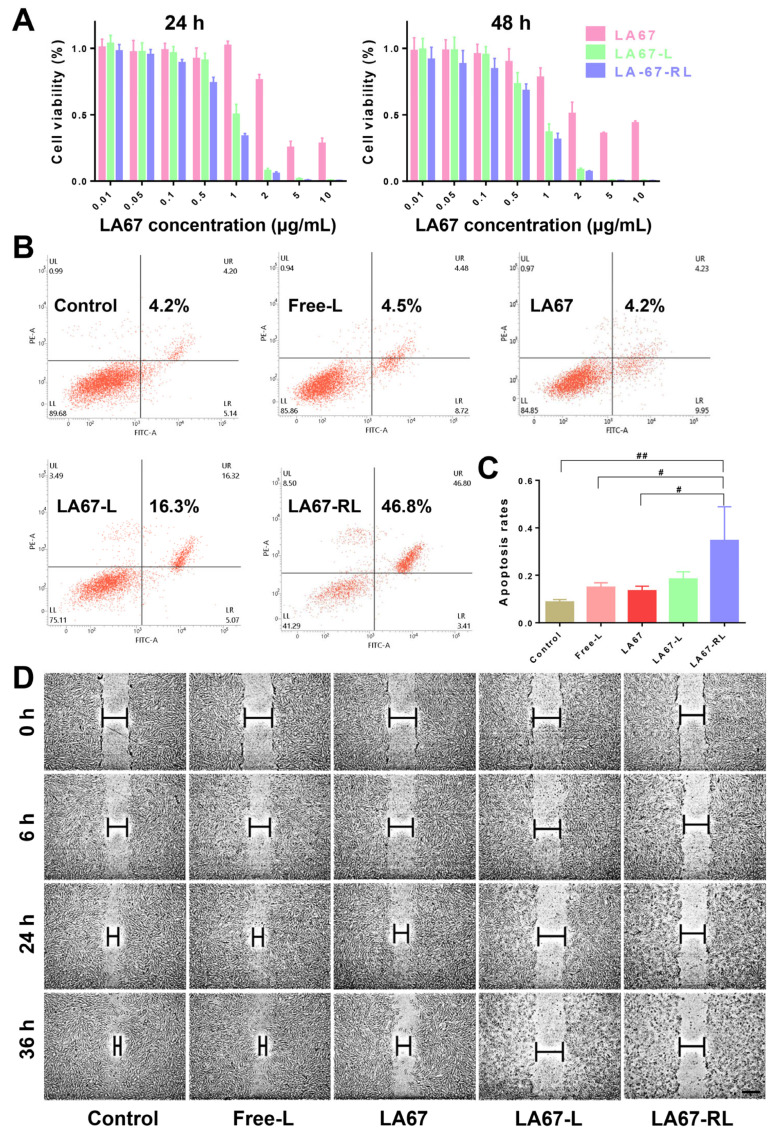
Inhibition, apoptosis, and migration. (**A**) Inhibitory capacity of LA67, LA67-L, and LA67-RL against KF cells proliferation (n = 6, Means ± SD). (**B**,**C**) Flow cytometry results of KF cells apoptosis and the percentage of early and late apoptosis after 24 h of treatment with LA67, LA67-L, and LA67-RL (n = 6, means ± SD), ^#^ *p* < 0.05, ^##^ *p* < 0.01. (**D**) Cell scratch healing rates during the 36 h (scale bar: 100 μm).

Small-molecule drugs typically enter tumor cells mainly through passive diffusion, while LNP can be incorporated into cells through various endocytosis pathways. To further investigate the inhibitory effect of LA67-RL, we examined the inhibitory effect of drug-loaded LA67-RL on KF cells proliferation and set a series of concentrations (0.01–10 μm/mL) and two timeframes (24 and 48 h). In vitro cytotoxicity of LA67-RL and free LA67 is shown in Figure 5A. The inhibitory effect of LA67-RL on KF cells was found to be concentration-dependent in the range of 0.1–10 μg/mL and action time. Cell viability was less than 10% in the LA 67-RL (LA67 concentration is 2 μg/mL) group at both 24 and 48 h.

#### 3.6.5. Cell Apoptosis

To examine the effect of LA67-RL on KF cells, annexin V-FITC/PI staining was used to assess KF cell apoptosis. As shown in Figure 5B,C, control and free-L groups did not significantly induce apoptosis in KF cells (4.2% and 4.5%), indicating that the blank material did not affect apoptosis, again demonstrating the safety of the carrier material. The free LA67 and LA67-L groups had the same apoptotic efficiency and generally induced a smaller proportion of apoptosis. As expected, the proportion of apoptotic cells in the LA67-RL group was significantly higher than that in the LA67 and LA67-L groups, which was 46.8%, indicating that LA67-RL can effectively promote the ability of LA67 to induce apoptosis of KF cells. RGD ligands could substantially increase the uptake of KF cells and enhance the LA67 delivery.

#### 3.6.6. Cell Migration

Keloids are formed due to excessive tissue repair following inflammation or minor injury and invasive growth into the surrounding area. To further evaluate the anti-tumor metastasis effect of LA67-RL, a cell scratch assay was used to test random cell migration. As shown in Figure 5D, scratches in the control and free-L groups healed gradually. The wound healing rates of the LA67, LA67-L, and LA67-RL groups were significantly slower within 36 h, indicating that LA67 generally inhibited the migration of KF cells. Particularly, scratches in LA67-RL group remained visible owing to the more significant cytotoxicity and specific inhibition of KF cell migration compared to the other groups. Importantly, LA67-RL exhibited a more substantial antimetastatic effect than the other formulations.

### 3.7. In Vivo Studies

#### 3.7.1. In Vivo Retention

**Figure 6:**
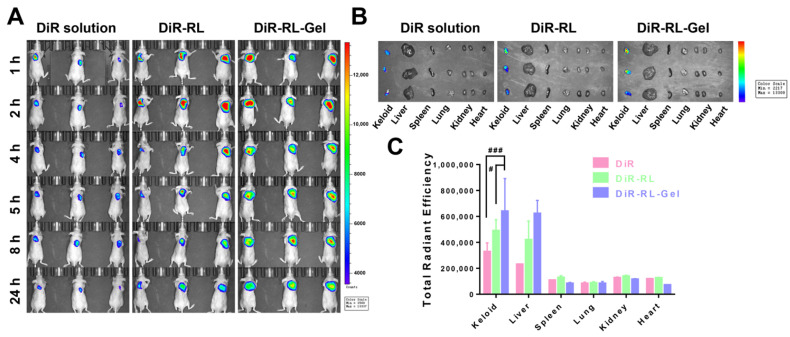
Biodistribution of free DiR solution, DiR-RL, and DiR-RL-Gel in vivo. (**A**) In vivo fluorescence images of keloid-bearing nude mice treated with DiR solution, DiR-RL, and DiR-RL-Gel. Images were taken at 2, 4, 6, 8, and 24 h after injection. (**B**) The fluorescence images of the excised keloids and major organs at 24 h after injection (n = 3, means ± SD). (**C**) The quantitative ROI analysis of the excised keloids and major organs at 24 h after injection (n = 3, means ± SD), ^#^ *p* < 0.05 and ^###^ *p* < 0.001.

Frequent administration may result in secondary damage and secondary infections. Therefore, it is crucial to construct a hydrogel with long-term mechanical stability and reduce the frequency of administration. Fluorescently labelled LNP hydrogels were injected subcutaneously into nude mice, and the in vivo retention of the hydrogels was measured by a small animal live imaging system. The results are shown in Figure 6A. At 1 h, the fluorescence intensity of the DiR solution group was significantly lower than that of the DiR-RL and DiR-RL-Gel groups, indicating that the solution group was leaking or being rapidly metabolized. After 5 h, the fluorescence intensity of the DiR-RL-Gel group was remarkably higher than that of the DiR-RL group, confirming the increased retention of the hydrogel in vivo. Meanwhile, the fluorescence range of the DiR-RL-Gel group gradually became more extensive, which means that the hydrogel underwent diffusion in vivo, followed by gradual metabolism.

#### 3.7.2. Biodistribution

To better understand the biodistribution and accumulation of LNP hydrogels in various organs in vivo, the nude mice were sacrificed at 24 h and the keloids and the major organs (heart, liver, spleen, lung, and kidney) were removed. Figure 6B,C shows ex vivo organ imaging, showing a similar trend to the results from the in vivo image. The keloid fluorescence intensity of nude mice treated with DiR-RL-Gel was 1.9- and 1.3-fold higher than that of nude mice treated with DiR solution and DiR-RL, respectively.

#### 3.7.3. In Vivo Anti-Keloid Effect

**Figure 7:**
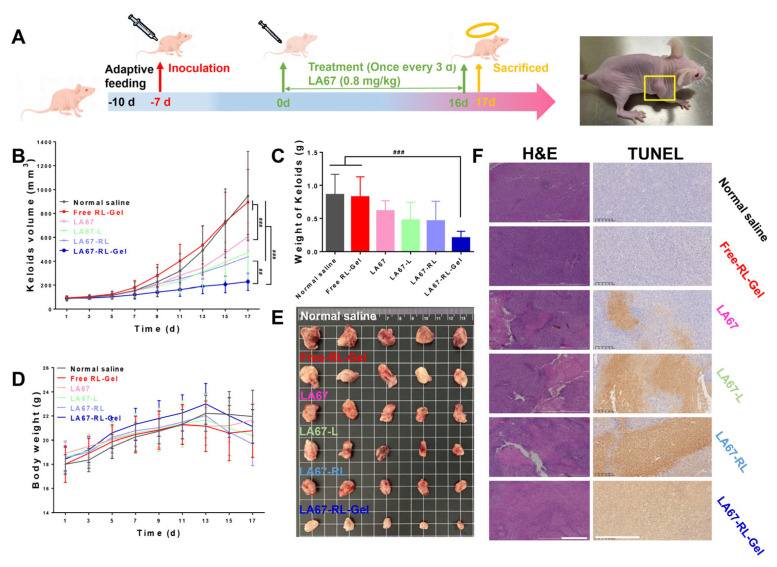
In vivo anti- keloid efficacy of LA67-RL-Gel in keloid-bearing nude mice. (**A**) A schematic illustration of the process and timeline of keloid-bearing nude mice. (**B**) Keloid volume of nude mice (n = 6, means ± SD), ^##^ *p* < 0.01 and ^###^ *p* < 0.001. (**C**) Weight of isolated keloids (n = 6, means ± SD), ^###^ *p* < 0.001. (**D**) Body weight of nude mice. (**E**) Image of isolated keloid tissues of nude mice. (**F**) H&E and TUNEL assay of keloid tissues isolated from nude mice (scale bar: 500 μm).

We investigated the anti-keloid effect of LA67-RL-Gel in vivo with BALB/c nude mice. Figure 7A shows the flow chart of animal experiments, including the model establishment and treatment process. The normal saline and free RL-Gel groups had the fastest keloid growth, and the surface skin of nude mice showed ulceration and bleeding at the end of the experiment, reflecting the necessary treatment of keloids. The LA67-RL-Gel group showed significantly slower keloid growth than LA67, LA67-L, and LA67-RL, demonstrating its potential to inhibit keloid growth (Figure 7B). This also proves once again the highly effective anti-keloid advantage of the LNP hydrogel. The weights and physical diagrams of the keloids are shown in Figure 7C,D. The results were consistent with Figure 7B. The body weight of the normal saline and free RL-Gel groups was minimal, and the body weight of the treated nude mice maintained a usual growth trend (Figure 7D). The body weight of nude mice in the LA67-RL-Gel group started to decrease after the 13th day of treatment, which may be due to the reduction of keloids (Figure 7E) and lower body weight, and also demonstrated the good in vivo anti-keloid growth effect.

Histological studies of isolated keloids were carried out. The H&E results showed proper fibroblast growth in the normal saline and free RL-Gel groups (Figure 7F). The LA67-RL-Gel group displayed many necrotic cells and a significant decrease in fibroblast density. TUNEL staining showed that the area of the apoptotic site in LA67-RL-Gel group was significantly larger than in other groups (Figure 7F), indicating that LA67-RL-Gel could significantly induce apoptosis and inhibit keloid growth.

#### 3.7.4. Anti-Keloid Mechanism

**Figure 8:**
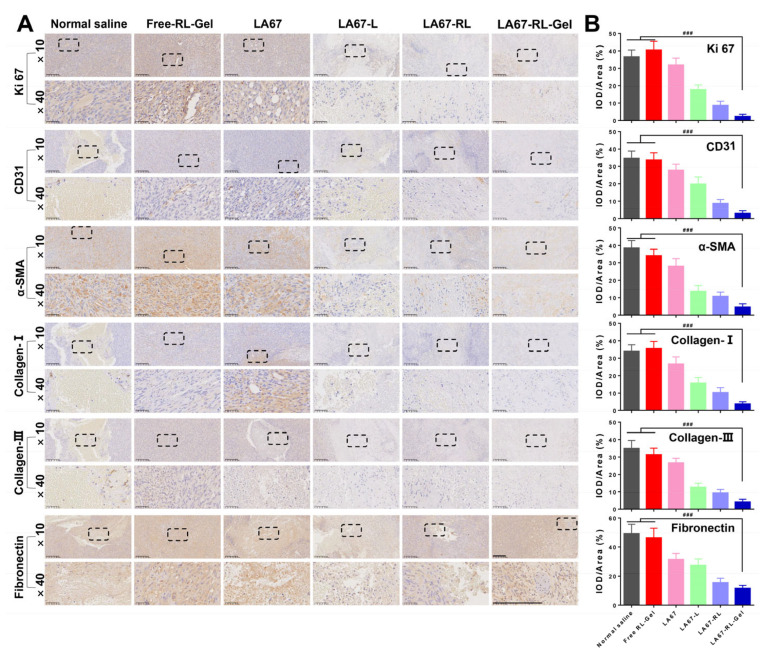
Immunocytochemistry results of isolated keloids. (**A**) Ki 67, CD31, α-SMA, collagen I, collagen III, and fibronectin (scale bar: 200 μm). The ×40 image is an enlargement of the dashed box in the ×10 image. (**B**) Statistical analysis of the data in (**A**) (n = 3, means ± SD), ^###^ *p* < 0.001.

To investigate the anti-keloid mechanism of LA67-RL-Gel, the regulatory effect of LA67-RL-Gel on the keloid microenvironment was studied. The results of immunohistochemistry are shown in Figure 8A,B.

Firstly, immunohistochemistry analysis was performed using anti-Ki 67 antibodies. Ki 67 is a marker of cell proliferation and is present during the active phase of mitosis. Ki 67 was significantly lower in the treatment groups (LA67, LA67-L, LA67-RL, and LA67-RL-Gel) compared to the normal saline and free RL-Gel groups, suggesting that LA67 inhibits cell proliferation. Among them, Ki 67 expression was the least in the LA67-RL-Gel group, demonstrating the outstanding in vivo anti-keloid effect of LA67-RL-Gel. Then, immunohistochemistry analysis was performed using anti-CD31 and α-Smooth muscle actin (α-SMA) antibodies. When a keloid grows, the angiogenic process becomes an essential tool for assessing disease progression and patient prognosis. CD31 is a vascular marker used to evaluate microvascular density at tumor sites. α-SMA, expressed on the cell surface, was observed to estimate the existence of newly generated vessels. The LA67-RL-Gel group had the least amount of CD31 and α-SMA, indicating that LA67-RL-Gel can reduce CD31 secretion, decrease α-SMA content, inhibit angiogenesis, and exert antifibrotic effects, thus treating keloids. Finally, the expression of collagen and fibronectin was further examined. Collagen and fibronectin are well known to be crucial components of the extracellular matrix, not only providing support for cell growth but also regulating cell behavior. They have also been extensively evaluated as a diagnostic marker for fibrosis. It was found that after LA67 treatment, Collagen-I, Collagen-III, and fibronectin levels were reduced in all groups, especially the LA67-RL-Gel group, which had the slightest expression. The above results indicated that LA67-RL-Gel has an excellent in vivo anti-keloid effect by modulating the keloid microenvironment and deserves further investigation and development.

#### 3.7.5. Biocompatibility and Tissue Cytotoxicity In Vivo

**Figure 9:**
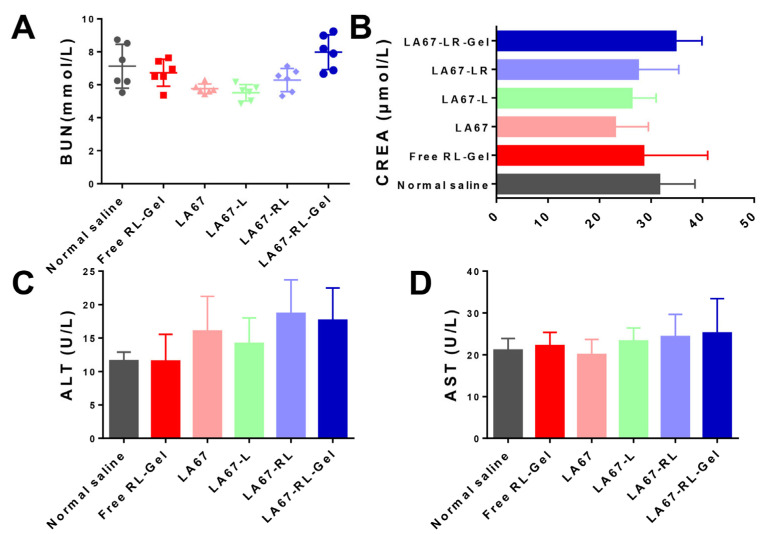
In vivo safety assessment. (**A**) BUN, (**B**) CREA, (**C**) ALT, and (**D**) AST levels in blood samples isolated from nude mice after treatment with normal saline, free RL-Gel, LA67, LA67-L, LA67-RL, and LA67-RL-Gel, respectively (n = 6, X ± SD).

High biocompatibility and low tissue toxicity of the as-prepared LA67-RL-Gel are essential for in vivo applications. The safety of the formulation was studied by blood biochemical analyses such as BUN, CREA, ALT, and AST. As shown in Figure 9, the blood concentration levels of BUN, CREA, ALT, and AST remained negligible among all groups. Our results indicated the excellent biocompatibility and biosafety of the LA67-RL-Gel for in vivo drug delivery.

## 4. Discussion

Keloids are a skin disease that cause life and mental stress to patients due to their severe aesthetic impact, characterized by excessive proliferation of fibroblasts and deposition of extracellular matrix leading to fibrosis [31]. This study aimed to explore an RGD-modified LNP hydrogel loaded with LA67 for treating keloids by peritumoral injection. Local injections of hydrogels offer an attractive approach because they can be given without intravenous injection, providing a more sustained release and high dose of therapeutic agents at the lesion, preventing prolonged blood circulation, and reducing side effects on normal tissues. LA67-RL-Gel gels were faster at 37 °C compared to the our team’s previous studies [31].

Triptolide is one of the main active ingredients of Tripterygium wilfordii Hook. F. Studies have shown that it has antioxidant, anti-rheumatoid, anti-Alzheimer’s, and anti-cancer properties [32]; however, triptolide has apparent hepatorenal toxicity. Through structural modification, the homologous compound LA67 was obtained, and the toxicity was significantly reduced. Nevertheless, LA67 has poor solubility in water, weak permeability, and poor bioavailability. Encapsulating hydrophobic drugs in nanocarriers has always been an excellent solution to overcome its low solubility and can also improve its pharmacological activity [33]. Therefore, in this study, we optimized an LNP formulation for encapsulating the hydrophobic drug LA67 to enhance its water solubility, bioavailability, and efficient delivery into the keloid environment. The hydrophobic drug loading in LNP and the release profile of encapsulated drugs in LNP bilayers are extremely dependent on the physicochemical properties of the drug and the LNP bilayer structure. DSPE-mPEG_2000_ is an amphiphilic phospholipid that enters the LNP bilayer through its hydrophobic portion (DSPE), and its hydrophilic portion (PEG) forms a shell around the liposome, which inhibits LNP adsorption by plasma proteins and uptake by the reticuloendothelial system [34].

The internalization process of nanocarriers by cells always involves two steps: surface binding on the cells and the following internalization into cells. LNP can increase the rate and amount of drug uptake by cells and has a sustained release effect on the drug, providing sustained drug release and cell killing at the pathological site. Due to the specificity of keloid sites, modification of nanocarriers by RGD peptides allows targeted neovascularization, improved cell adhesion, and increased uptake for more effective, precise, and safe treatment [35]. As RGD competes with integrin ligands for integrin receptors, it plays an adjuvant therapeutic role by inhibiting the corresponding signaling and maximizing the effect of killing KF cells. The experimental results also showed that RGD-modified LNP significantly increased the cellular uptake of LA67 by active targeting. Local administration can significantly reduce the systemic toxicity and side effects of drugs. Pozharani et al. reported that metronidazole was added directly to a gel composed of P407 and P188 and was found to release 90% in the first 3 h [36]; our gel releases 71.4 ± 0.7% at 24 h. Encapsulation of the drug in LNP reduces the rate of drug release. The slow drug release is beneficial to the full uptake of cells and improves the bioavailability. In situ hydrogels prolong the retention time in vivo, and continuously deliver drugs at the keloid site, increasing the anti-keloid effect.

Collagen deposition plays an important role in the development of fibrosis [37]. Fibroblasts secrete collagen, which causes excess extracellular matrix and leads to keloid formation. In addition, the presence of fibroblastic lesions is an important pathological feature of keloids. When keloids occur, Ki 67 increases significantly, promoting cell proliferation. At the same time, the keloid microenvironment overexpression of α-SMA, and secretion of a large number of extracellular matrix components occurs (mainly denotes collagen Ⅰ, collagen Ⅲ, and fibronectin). Collagen deposition accelerates the development of tissue fibrosis. Next, a large amount of CD31 is secreted in the keloid microenvironment to accelerate keloid formation. In vitro and in vivo studies have shown that LA67-RL-Gel plays a crucial role in treating keloids. LA67-RL-Gel promotes cell apoptosis; inhibits cell proliferation; reduces α-SMA content; decreases collagen I, collagen III, and fibronectin deposition; inhibits angiogenesis; and modulates the keloid microenvironment, ultimately exerting anti-keloid effects.

## 5. Conclusions

In this study, we developed a LA67 LNP-loaded thermo-sensitive hydrogel for treating keloids via peritumoral injection and explored the anti-keloid mechanism. LA67-RL has a uniform particle size, is simple to prepare, improves the solubility of LA67, and has a high encapsulation efficiency. Due to the presence of RGD, this LNP efficiently promotes KF cell uptake, induces KF cell apoptosis, and inhibits cell migration. LA67-RL-Gel significantly prolongs the in vivo retention time, providing a more sustained release and high dose of therapeutic agents at the keloid, demonstrating outstanding in vivo anti-keloid effects. In conclusion, the LA67-RL-Gel prepared in this experiment has a high safety profile. It can significantly increase the therapeutic effect of LA67 on keloids, which is expected to be a new treatment strategy for keloids. In the future, our team will further investigate the anti-keloid mechanism of LA67-RL-Gel, which may allow for translating this formulation to clinical practice.

## Figures and Tables

**Table 1:** Variables for optimization of thermo-sensitive hydrogels using BBD.

Independent Variables		Range	
		Minimum	Maximum
X_1_	P407 (%)	18	24
X_2_	P188 (%)	1	3
Responses		Target	
Y	Gelation time (s)	Minimum	

**Table 2:** Research on hydrogel characteristics based on BBD (n = 3, means ± SD).

	Independent Variables		Response Variable
Run	X_1_ (%)	X_2_ (%)	Y (S)
1	25.3	2.0	36.0 ± 3.2
2	21.0	2.0	11.0 ± 2.1
3	21.0	2.0	9.1 ± 1.2
4	16.8	2.0	23.9 ± 2.1
5	21.0	3.4	14.1 ± 2.3
6	24.0	3.0	29.9 ± 3.3
7	21.0	2.0	8.1 ± 1.1
8	24.0	1.0	32.9 ± 4.3
9	21.0	2.0	10.3 ± 2.0
10	18.0	3.0	29.8 ± 3.4
11	21.0	2.0	10.2 ± 1.0
12	18.0	1.0	30.3 ± 3.2
13	21.0	0.6	13.0 ± 2.1

**Table 3:** The statistical analysis of variations in the response variables.

Source	Sum of Squares	df	Mean Squares	F-Value	*p*-Value
Model	1177.7	5	235.5	8.5	0.0069 significant
A-P407	49.9	1	49.9	1.8	0.2218
B-P188	0.3	1	0.3	0	0.9182
X_1_X_2_	2.3	1	2.3	0.1	0.7840
X_1_	1078.3	1	1078.3	38.9	0.0004
X_2_	122.7	1	122.7	4.4	0.0734
Residual	194.0	7	27.7		
Lack of Fit	188.8	3	62.9	48.4	0.0013
Pure Error	5.2	4	1.3		
Cor Total	1371.7	12			

## Data Availability

The data presented in this study are available in this article.

## Abbreviations

RGD, Arg-Gly-Asp; LNP, lipid nanoparticle; BBD, Box–Behnken design; KF, keloid fibroblast; FT-IR, Fourier transform infrared spectroscopy; PDI, polydispersity indexes; EE, encapsulation efficiency; CLSM, confocal laser scanning microscopy; P407, Poloxamer 407; P188, Poloxamer 188.
